# Associations of plasma high-sensitivity C-reactive protein concentrations with all-cause and cause-specific mortality among middle-aged and elderly individuals

**DOI:** 10.1186/s12979-019-0168-5

**Published:** 2019-11-05

**Authors:** Zhi-Hao Li, Wen-Fang Zhong, Yue-Bin Lv, Virginia Byers Kraus, Xiang Gao, Pei-Liang Chen, Qing-Mei Huang, Jin-Dong Ni, Xiao-Ming Shi, Chen Mao, Xian-Bo Wu

**Affiliations:** 1 Department of Epidemiology, School of Public Health, Southern Medical University, Guangzhou, 510515 Guangdong China; 2 National Institute of Environmental Health, Chinese Center for Disease Control and Prevention, Beijing, China; 3 Duke Molecular Physiology Institute and Division of Rheumatology, Department of Medicine, Duke University School of Medicine, Durham, North Carolina USA; 4 Department of Nutritional Sciences, Pennsylvania State University, University Park, PA USA; 5 Department of Epidemiology and Biostatistics, Dongguan Key Laboratory of Environmental Medicine, School of Public Health, Guangdong Medical University, Dongguan, Guangdong China

**Keywords:** High-sensitivity C-reactive protein, Cardiovascular mortality, Cancer mortality, All-cause mortality

## Abstract

**Background:**

The association of high-sensitivity C-reactive protein (hsCRP) with mortality is controversial. We aimed to investigate the associations of hsCRP concentrations with the risks of all-cause and cause-specific mortality and identify potential modifying factors affecting these associations among middle-aged and elderly individuals.

**Methods:**

This community-based prospective cohort study included 14,220 participants aged 50+ years (mean age: 64.9 years) from the Health and Retirement Study. Cox proportional hazard models were employed to estimate the associations between the hsCRP concentrations and the risk of all-cause and cause-specific mortality with adjustment for sociodemographic and lifestyle factors, self-reported medical history, and other potential confounders.

**Results:**

In total, 1730 all-cause deaths were recorded, including 725 cardiovascular- and 417 cancer-related deaths, after an 80,572 person-year follow-up (median: 6.4 years; range: 3.6–8.1 years). The comparisons of the groups with the highest (quartile 4) and lowest (quartile 1) hsCRP concentrations revealed that the adjusted hazard ratios and 95% confidence intervals were 1.50 (1.31–1.72) for all-cause mortality, 1.44 (1.13–1.82) for cardiovascular mortality, and 1.67 (1.23–2.26) for cancer mortality. The associations between high hsCRP concentrations and the risks of all-cause, cardiovascular, and cancer mortality were similar in the men and women (*P* for interaction > 0.05).

**Conclusions:**

Among middle-aged and older individuals, elevated hsCRP concentration could increase the risk of all-cause, cardiovascular, and cancer mortality in men and women.

## Introduction

Inflammation plays a key role in the initiation and progression of atherosclerosis and other diseases (e.g., hypertension and stroke) processes [1–3]. High-sensitivity C-reactive protein (hsCRP) is an acute-phase protein produced in the liver during the inflammatory cascade [4]. Although multiple biomarkers of inflammation exist, hsCRP remains an extensively used marker of inflammation because the concentrations are relatively stable and its detection is relatively inexpensive and highly sensitive [5].

Numerous studies have investigated the associations between hsCRP concentrations and mortality. For instance, recently, elevated hsCRP concentrations were shown to be associated with the risk of all-cause mortality in both men and women in many [6–9], but not all studies [10]. Two other prospective studies were conducted in the United States and indicated that high CRP levels were associated with significantly increased risks of all-cause and CVD-related mortality [11, 12]. However, findings regarding the effect of hsCRP concentrations on cancer mortality remain controversial. Several studies have suggested that an increased risk of cancer mortality is associated with elevated hsCRP concentrations [9, 10, 13–16]. In contrast, a recent prospective study failed to identify a similar association [17]. These inconsistent results may be attributed to age and sample size differences in the populations studied [9]. Moreover, previous studies have suggested a reduced capacity to respond to inflammation with age, which caused CRP level to be more detrimental for mortality among the older than younger [18, 19]. Another study indicated that estrogens have negative effects on inflammatory cell migration and inflammatory marker production [20], resulting in gender difference in association between hsCRP and mortality. However, little is currently known about whether the associations of hsCRP concentrations with mortality vary between men and women, and vary by subgroups of age in population studies.

Therefore, using community-based cohort data from the Health and Retirement Study (HRS), we aimed to investigate the associations of hsCRP concentrations with all-cause and cause-specific mortality and to identify potential modifying factors affecting these associations, among middle-aged and older individuals (≥50 years).

## Methods

### Design, study setting, and participants

This study was performed as part of the HRS, an ongoing, nationally representative community-based prospective cohort study of middle-aged and elderly Americans. Details regarding the participants and study design have been previously reported [21]. In brief, the participants were interviewed in 1992 and every 2 years thereafter; five additional waves of participants were added in phases between 1994 and 2014. Starting in 2006, an enhanced face-to-face interview that included biomarker assessment was implemented as part of the HRS (*http://hrsonline.isr.umich.edu*). For the present analysis, we used only the data for participants aged ≥50 years from 2006 to 2014. Participants with missing hsCRP concentration data, hsCRP concentrations > 10 mg/L, or cancer at baseline were excluded. In total, 14,220 people (6118 men and 8102 women) were eligible. A flowchart of participant enrollment is shown in Additional file 1: Figure. S1. Ethical approval for the HRS was obtained from the University of Michigan Institutional Review Board; all respondents provided written informed consent.

### Measurement of plasma hsCRP concentrations

HsCRP concentrations were measured with an enzyme-linked immunosorbent assay using a dried blood spot (DBS) at the University of Vermont [22]. The hsCRP concentrations had a lower limit of detection of 0.035 mg/L, with within-assay and between-assay variability of 8.1 and 11.0%, respectively [22].

### Assessment of deaths

Deaths were ascertained in each cohort via data from the National Death Index and exit interviews with family members. Previous HRS analyses showed a rate of death validation greater than 99% [21]. Death due to a heart, circulatory or blood condition was classified as cardiovascular mortality. Cancer mortality was indicated if the cause of death was recorded as cancer. We calculated the person-time (in months) from the return of the baseline questionnaire until the date of death or December 31, 2014, whichever occurred first.

### Covariates

Several of the potential confounders included in the current study were selected based on previous studies [9, 23]. The covariates included sociodemographic information (age, sex, ethnicity, educational levels, and household income), lifestyle factors (current smoking status, alcohol consumption, regular exercise and body mass index [BMI]), clinical measures (concentrations of total cholesterol [TC], high-density lipoprotein cholesterol [HDL-C] and hemoglobin A1c [HbA1c]), the 8-question Center for Epidemiologic Studies Depression Scale (CES-D 8) score, self-reported medical history (hypertension, diabetes, heart disease, stroke, psychological problems and pulmonary disorders), and limitations in any of five activities of daily living (ADLs): bathing, getting in and out of bed, dressing, walking across a room, and eating. Ethnicity, education levels and household income were measured by self-report using the following categories: white, black, and other; < 12, 12–15, or > 15 years; ≤$20,000, $20,001–$50,000, or > $50,000, respectively. We dichotomized current alcohol consumption as drinking (one or more drinks per day) versus not drinking. BMI was defined as by weight in kilograms divided by the square of height in meters. All covariate data were collected from the structured questionnaire and biochemistry tests administered at baseline (available at http://hrsonline.isr.umich.edu).

### Statistical analysis

HsCRP concentrations were classified as belonging to quartile 1 (Q1, < 0.86 mg/L), quartile 2 (Q2, 0.86–1.74 mg/L), quartile 3 (Q3, 1.75–3.59 mg/L), or quartile 4 (Q4, > 3.59 mg/L). Baseline tables were generated using descriptive statistics (means and standard deviations [SDs] or %) stratified by hsCRP quartiles. Kaplan-Meier curves were generated for the quartiles of hsCRP concentrations, and log-rank tests were used to compare different groups. Cox proportional hazards regression models were applied to estimate hazard ratios (HRs) with 95% confidence intervals (95% CIs) for mortality according to the hsCRP quartiles, using the lowest quartile (Q1) as the reference group. We also evaluated the HRs of all-cause and specific-cause mortality per each 1 mg/L increase in the hsCRP concentration. The Cox proportional hazards assumptions were assessed with Schoenfeld residual plots, and no major violation of the assumptions was observed. Two models with adjustments for different variables were used. The baseline model (model 1) tested the association between the hsCRP concentrations and mortality and controlled for age and sex, while the multivariable-adjusted model (model 2) further adjusted for ethnicity (white, black, or other), education level (< 12, 12–15, or > 15 years), household income (≤20,000$, 20,001$-50,000$, or > 50,000$), BMI (continuous variable), smoking status (current smoker or nonsmoker), alcohol consumption (current drinker or nondrinker), regular exercise (yes or no), HDL-C (continuous variable), TC (continuous variable), and HbA1c (continuous variable). Moreover, we examined the extent to which the associations between each 1 mg/L increase in the hsCRP concentration and all-cause and cause-specific mortality were explained by the mediators (hypertension, heart disease, stroke, diabetes, pulmonary disorder, CES-D 8 score, psychological problems and limitations in ADLs). To correct for missing values and reduce the potential for inferential bias, we imputed missing covariate data using multiple imputation methods [24].

Effect modifications of the associations between each 1 mg/L increase in hsCRP concentration and all-cause and cause-specific mortality by sex (men or women), age (< 65 or ≥ 65 years), BMI (obese [> 30 kg/m^2^] or nonobese [≤30 kg/m^2^]), current smoking status (smoker or nonsmoker), and current alcohol consumption (drinker or nondrinker) were assessed by computing likelihood ratios comparing the statistical fit of models with and without interaction terms in the fully adjusted model.

We conducted several sensitivity analyses, such as excluding all participants who died during the first 2 years of follow-up, to determine the robustness of our primary findings; individuals were stratified by tertiles, quintiles and clinical categories of hsCRP concentrations (< 1, 1–3, or > 3 mg/L) [25]. Analyses were performed with R software version 3.5.1 (R Foundation for Statistical Computing, Vienna, Austria); a two-tailed *P* value < 0.05 was considered statistically significant.

## Results

### Baseline characteristics

Table 1 presents the characteristics of participants stratified by hsCRP quartiles at baseline. The mean age was 64.9 years, and 57.0% of the participants were women. The median concentration of hsCRP was 2.02 mg/L. Compared with participants with lower hsCRP concentrations, those with higher hsCRP concentrations were more likely to be women, black, less educated, and current smokers; those with higher hsCRP concentrations were also more likely to have a lower household income and higher BMI. The prevalence rates of hypertension, diabetes, pulmonary disorders, heart disease, stroke, psychological problems and limitations in ADLs increased with increasing quartiles of hsCRP (Table 1).

**Table 1:** Baseline characteristics of participants stratified by high-sensitivity C-reactive protein concentration quartiles

Characteristics	Overall	HsCRP concentration quartiles (mg/L)			
		Q1 (< 0.86)	Q2 (0.86–1.74)	Q3 (174–3.59)	Q4 (> 3.59)
No. of participants	14,220	3546	3556	3551	3567
Age, years	64.9 (10.3)	64.6 (10.5)	65.3 (10.4)	65.0 (10.3)	64.8 (10.1)
Women (%)	8102 (57.0)	1825 (51.5)	1878 (52.8)	2048 (57.7)	2351 (65.9)
Race (%)					
White	10,628 (74.7)	2701 (76.2)	2752 (77.4)	2649 (74.6)	2526 (70.8)
Black	2433 (17.1)	517 (14.6)	497 (14.0)	630 (17.7)	789 (22.1)
Other	1159 (8.2)	328 (9.2)	307 (8.6)	272 (7.7)	252 (7.1)
Household income (%), $					
< 20,000	3489 (24.5)	741 (20.9)	772 (21.7)	907 (25.5)	1069 (30.0)
20,001-50,000	4602 (32.4)	1077 (30.4)	1168 (32.8)	1190 (33.5)	1167 (32.7)
> 50,000	6129 (43.1)	1728 (48.7)	1616 (45.4)	1454 (40.9)	1331 (37.3)
Education levels, years (%)					
< 12	2455 (17.3)	488 (13.8)	569 (16.0)	671 (18.9)	727 (20.4)
12–15	7907 (55.6)	1855 (52.3)	1959 (55.1)	2020 (56.9)	2073 (58.1)
> 15	3858 (27.1)	1203 (33.9)	1028 (28.9)	860 (24.2)	767 (21.5)
BMI, kg/m^2^	28.37 (5.74)	25.97 (4.61)	27.56 (4.85)	29.11 (5.44)	30.81 (6.68)
Current smoker (%)	7963 (56.0)	1835 (51.7)	1949 (54.8)	2050 (57.7)	2129 (59.7)
Current drinker (%)	5288 (37.2)	1501 (42.3)	1414 (39.8)	1279 (36.0)	1094 (30.7)
Regular exercise (%)	11,675 (82.1)	3071 (86.6)	3035 (85.3)	2873 (80.9)	2696 (75.6)
HDL-C, mg/dL	54.5 (16.1)	56.5 (16.8)	54.8 (16.1)	53.8 (16.0)	52.9 (15.4)
HbA1c, %	5.9 (1.1)	5.7 (0.9)	5. 8 (1.0)	5.9 (1.1)	6.0 (1.2)
TC, mg/dL	201.1 (42.6)	197.3 (41.3)	200.7 (42.2)	202.1 (43.2)	204.3 (43.4)
CES-D 8 score	1.5 (2.0)	1.3 (1.9)	1.4 (1.9)	1.6 (2.1)	1.7 (2.1)
Pulmonary disorder (%)	773 (5.4)	123 (3.5)	150 (4.2)	221 (6.2)	279 (7.8)
Heart disease (%)	2853 (20.1)	657 (18.5)	706 (19.9)	718 (20.2)	772 (21.6)
Stroke (%)	902 (6.3)	189 (5.3)	228 (6.4)	219 (6.2)	266 (7.5)
Psychological problems (%)	2195 (15.4)	498 (14.0)	483 (13.6)	564 (15.9)	650 (18.2)
Hypertension (%)	7627 (53.6)	1587 (44.8)	1861 (52.3)	1996 (56.2)	2183 (61.2)
Diabetes (%)	2790 (19.6)	598 (16.9)	637 (17.9)	703 (19.8)	852 (23.9)
Limitation in ADLs (%)	1031 (7.3)	228 (6.4)	234 (6.6)	248 (7.0)	321 (9.0)

Values are expressed as the mean (standard deviation) or number (percentage);

*ADLs* Activities of daily living, *BMI* Body mass index, *CES-D 8 score* The 8-question Center for Epidemiologic Studies Depression Scale, *HbA1c* Hemoglobin A1c, *HDL-C* High-density lipoprotein cholesterol, *TC* Total cholesterol

### Plasma hsCRP concentrations and mortality

**Fig. 1:**
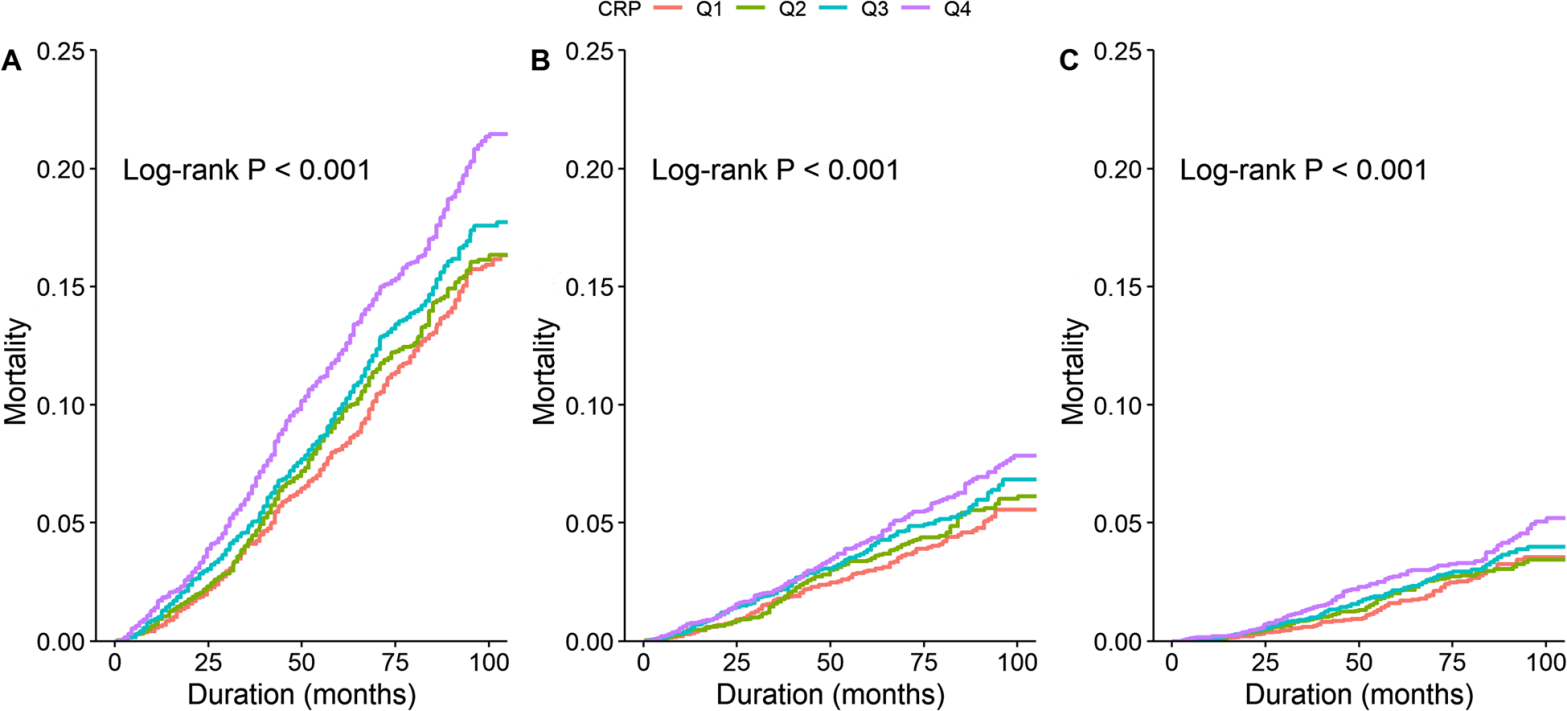
Kaplan-Meier curves for all-cause, cardiovascular and cancer mortality stratified by baseline high-sensitivity C-reactive protein concentration quartiles. **(a)** Kaplan-Meier curves of all-cause mortality; **(b)** Kaplan-Meier curves of cardiovascular mortality; **(c)** Kaplan-Meier curves of cancer mortality. If hsCRP < 0.86 mg/L, quartile 1 (Q1); if hsCRP ≤1.74 mg/L, quartile 2 (Q2); if hsCRP ≤3.59 mg/L, quartile 3 (Q3); and if hsCRP > 3.59 mg/L, quartile 4 (Q4)

During a total of 80,572 person-years of follow-up (median follow-up: 6.4 years, interquartile range: 3.6–8.1 years), 1730 deaths were recorded, including 725 from cardiovascular diseases and 417 from cancer. Rates of all-cause, cardiovascular and cancer mortality increased in association with increases in hsCRP assessed as quartiles (Fig. 1).

**Fig. 2:**
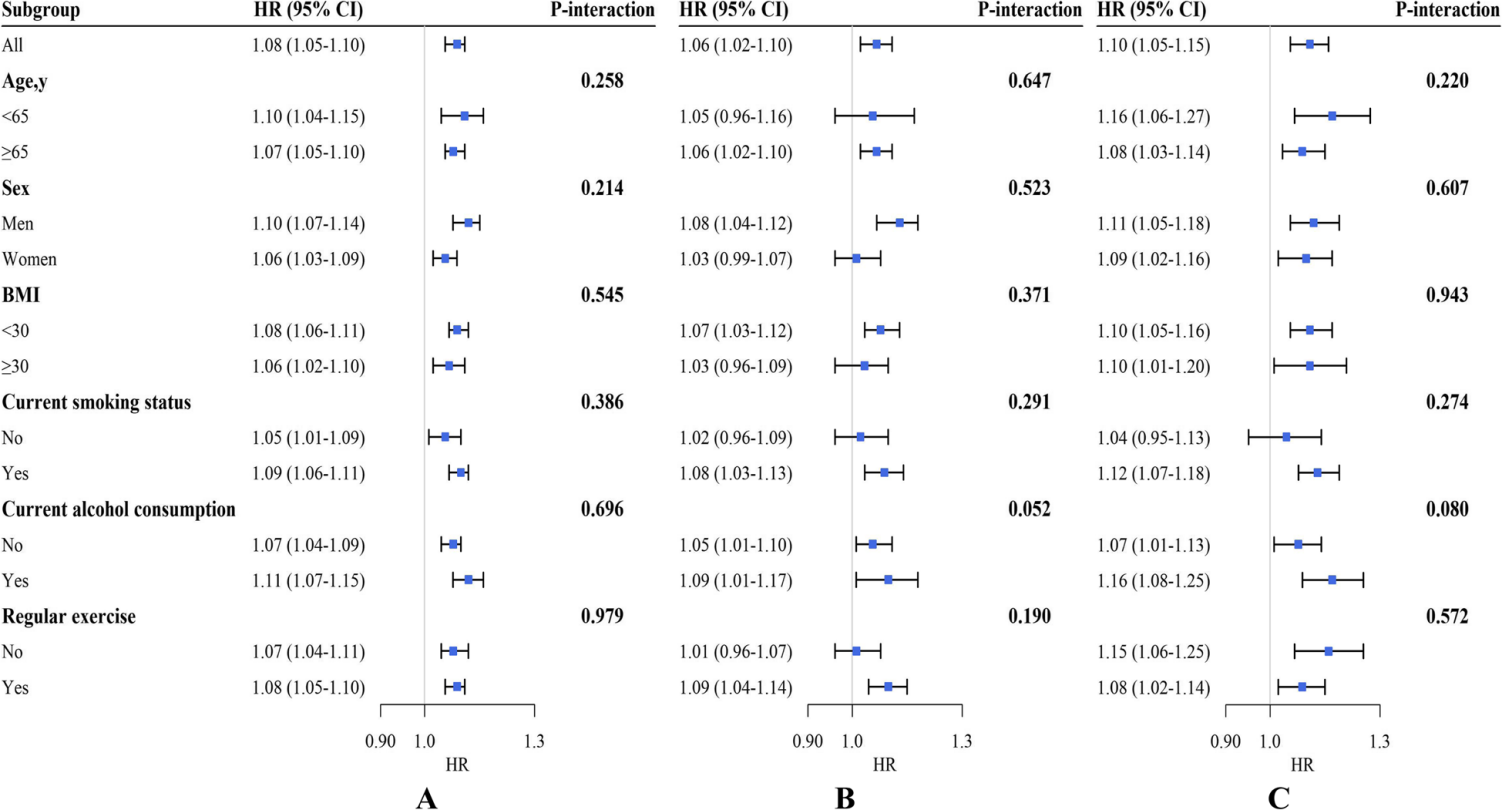
Subgroup analyses for the hazard ratio of all-cause (**a**), cardiovascular (**b**) and cancer mortality (**c**) for each 1 mg/L increase in hsCRP concentrations. Adjusted for age, sex, race, educational level, current smoking status, alcohol consumption, regular exercise, body mass index (BMI), household income, total cholesterol (TC) concentration, high density lipoprotein cholesterol (HDL-C) concentration, hemoglobin A1c (HbA1c), CES-D 8 score, hypertension, heart disease, stroke, diabetes, pulmonary disorder, psychiatric problems, and limitations in activities of daily living (ADLs)

The multivariable-adjusted HRs (95% CIs) of all-cause mortality with the lowest quartile (Q1) of hsCRP as the reference were 1.50 (1.31–1.72) for the highest quartile (Q4) (*P* for trend < 0.001). The multivariable-adjusted HRs (95% CIs) of cardiovascular and cancer mortality using the Q1 of hsCRP as the reference were 1.44(1.13–1.82) and 1.67 (1.23–2.26) for Q4, respectively (all *P* for trend < 0.001) (Table 2). Additionally, evaluating the risks of all-cause, cardiovascular, and cancer mortality associated with each 1 mg/L increase in hsCRP concentrations revealed multivariable-adjusted HRs (95% CIs) of 1.08 (1.05–1.10), 1.06 (1.02–1.10), and 1.10 (1.05–1.15), respectively (Fig. 2). Moreover, Additional file 1: Table S1 shows the role played by the potential mediators (hypertension, heart disease, stroke, diabetes, pulmonary disorder, CES-D 8 score, psychological problems and limitations in ADLs) in the association between the hsCRP concentrations and mortality. However, these associations between the hsCRP concentrations and all-cause, cardiovascular and cancer mortality were minimally explained by the mediators included in the model (Additional file 1: Table S1).

**Table 2:** HRs (95% CI) for all-cause, cardiovascular and cancer mortality stratified by baseline high-sensitivity C-reactive protein concentration quartiles

HsCRP quartiles	All-cause mortality		Cardiovascular mortality		Cancer mortality	
	Model 1^**a**^	Model 2^**b**^	Model 1	Model 2	Model 1	Model 2
No. of participants	14,220		14,220		14,220	
Person-years at risk	80,572		80,572		80,572	
No. of events	1730		608		351	
Q1	1.00 (reference)	1.00 (reference)	1.00 (reference)	1.00 (reference)	1.00 (reference)	1.00 (reference)
Q2	1.02 (0.88–1.17)	1.06 (0.92–1.22)	1.07 (0.84–1.36)	1.09 (0.82–1.33)	1.05 (0.76–1.44)	1.07 (0.78–1.48)
Q3	1.17 (1.02–1.35)*	1.18 (1.03–1.36)*	1.28 (1.01–1.62)*	1.26 (1.00–1.57)	1.23 (0.90–1.69)	1.23 (0.90–1.69)
Q4	1.59 (1.40–1.82)***	1.50 (1.31–1.72)***	1.62 (1.29–2.04)***	1.44 (1.13–1.82)**	1.71 (1.27–2.29)***	1.67 (1.23–2.26)**
*P* for trend	< 0.001	< 0.001	< 0.001	< 0.001	< 0.001	< 0.001

^**a**^Model 1: adjusted for age and sex

^**b**^Model 2: adjusted for age, sex, race, educational level, current smoking status, alcohol consumption, regular exercise, body mass index (BMI), household income, total cholesterol (TC) concentration, high density lipoprotein-cholesterol (HDL-C) concentration, hemoglobin A1c (HbA1c) at the endCES-D 8 score, hypertension, heart disease, stroke, diabetes, pulmonary disorder, psychiatric problems, and limitations in activities of daily living (ADLs)

* *P* < 0.05; ** *P* < 0.01; *** *P* < 0.001

### Subgroup analyses

Subgroup analysis by sex showed no evidence of a significant difference between men and women (all *P* for interaction > 0.05) regarding the associations of hsCRP concentrations with all-cause, cardiovascular and cancer mortality (Fig. 2). Additionally, we found no significant interaction effects for age group (≥65 years, and < 65 years), current smoking status (smoker or nonsmoker), current alcohol consumption (drinker or nondrinker), regular exercise (yes or no), or BMI (< 30 or ≥ 30 kg/m^2^) (all *P* for interactions > 0.05).

### Sensitivity analyses

Sensitivity analyses, excluding participants who died in the first 2 years of follow-up, did not notably alter the findings for all-cause, cardiovascular or cancer mortality (Additional file 1: Table S2). Moreover, the associations remained unchanged when individuals were divided into tertiles (Additional file 1: Table S3), quintiles (Additional file 1: Table S4) or clinical categories (Additional file 1: Table S5) based on hsCRP concentrations.

## Discussion

In this community-based prospective cohort study, higher plasma hsCRP concentrations were associated with increased risks of all-cause, cardiovascular and cancer mortality among middle-aged and older individuals, even after adjustment for several potential confounders. Specifically, the associations of hsCRP concentrations with all-cause, cardiovascular and cancer mortality did not differ substantially when participants were stratified by sex, age groups, BMI, regular exercise, current smoking status, or current alcohol consumption.

Our findings confirm the results of previous studies that showed positive associations between plasma hsCRP concentrations and the risks of all-cause and cardiovascular mortality [8, 13, 26]. A possible explanation for this phenomenon is that atherosclerosis is a chronic inflammatory process in which immune mechanisms interact with metabolic risk factors to initiate, propagate, and activate arterial lesions [27, 28]. In addition, the associations of hsCRP concentrations with diseases (e.g., cardiovascular disease [8, 29], diabetes [30], and dementia [31]) and the increased risks of mortality in patients with higher hsCRP concentrations and a variety of conditions, such as chronic obstructive pulmonary disease (COPD) [32] and stroke [33], likely form the basis for this finding. Furthermore, consistent with several previous studies [10, 13–17], our study showed that elevated hsCRP concentrations were associated with an increased risk of cancer mortality. A potential explanation for this finding is that approximately 15% of cancer worldwide is considered related to chronic infections [34] through mechanisms involving chronic local inflammation leading to DNA damage and mutagenesis [35].

According to some studies, hsCRP concentrations are more strongly associated with the risks of all-cause and cause-specific mortality in men than in women [10, 14, 36]. These studies revealed that the ability of hsCRP concentrations to predict mortality appeared to be dependent on sex. However, the underlying mechanism remains unclear. One important potential explanation is the effect modification of the association between hsCRP concentrations and mortality by female hormones. As a previous study reported [37], the sex-specific effect of elevated hsCRP concentrations on the risk of mortality is correlated with the concentrations of endogenous reproductive hormones. In contrast, the effects of hsCRP concentrations on mortality were similar in men and women in our study. This finding may be due to our inclusion of participants who were 50 years of age or older, which means that most of the women included in this study were peri- or postmenopausal, reducing the sex-specific effect of endogenous reproductive hormones on the association between hsCRP concentrations and mortality.

In our study, the association of hsCRP concentrations and mortality appeared to be similar in participants aged < 65 years and those aged ≥65 years, consistent with a previous study [14]. Moreover, obesity [38, 39] and alcohol consumption [40] are associated with a proinflammatory state, but the association between hsCRP concentrations and mortality was not modified by obesity and alcohol consumption in this study. Smoking is one of the most important contributors to elevated hsCRP concentrations and one of the most important risk factors for mortality [38]. However, the association between hsCRP concentrations and mortality, although slightly stronger in current smokers, was still notable in nonsmokers.

### Strengths and limitations

The strengths of this study are its population-based, prospective design, the large middle-aged and older groups, the adjustments for several identified and potential confounders, and the robust results of the subgroup and sensitivity analyses. Additionally, we examined cause-specific mortality, including cancer and cardiovascular mortality.

However, our study has several potential limitations that should be noted. First, the measurements of plasma hsCRP concentrations were single baseline examinations and may not accurately reflect the long-term plasma hsCRP statuses of the participants. Multiple plasma hsCRP measurements would have reduced the variability and enabled us to examine changes in plasma hsCRP concentrations; however, measuring hsCRP concentrations longitudinally in epidemiological studies is impractical and expensive. Second, data on statin use and estrogen therapy, which affect hsCRP concentrations, were not included in our analysis. The relationships of estrogen therapy and statin use with hsCRP concentrations and mortality risk should be further explored in a large population. Finally, although we carefully adjusted for several confounders, such as sociodemographic characteristics and lifestyle factors, the potential for residual confounders, such as other unmeasured or unknown covariates, likely remained.

## Conclusion

This study demonstrated that an elevated plasma hsCRP concentration is associated with the risk of all-cause, cardiovascular and cancer mortality in middle-aged and elderly American individuals. The associations of hsCRP concentrations with all-cause, cardiovascular and cancer mortality did not differ substantially between the sexes in individuals aged ≥50 years.

## Supplementary information

**Additional file 1: Figure. S1.** Flowchart of the participant enrolment. **Table S1.** Role of potential mediators in explaining the association between each 1 mg/L increase in the hsCRP concentration and all-cause, cardiovascular and cancer mortality. **Table S2.** Multivariable hazard ratios (HR [95% CI]) of mortality by quartiles of high-sensitivity C-reactive protein after excluding deaths during the first two years of follow-up. **Table S3.** Multivariable hazard ratios (HR [95% CI]) of mortality by tertiles of high-sensitivity C-reactive protein. **Table S4.** Multivariable hazard ratios (HR [95% CI]) of mortality by quintiles of high-sensitivity C-reactive protein. **Table S5.** Multivariable hazard ratios (HR [95% CI]) of mortality by clinical categories of high-sensitivity C-reactive protein.

## Data Availability

The Health and Retirement Study data are available to registered users at http://hrsonline.isr.umich.edu/index.php.

## Abbreviations

95% CIs: 95% Confidence intervals
ADLs: Activities of daily living
BMI: Body mass index
CES-D 8: The 8-question Center for Epidemiologic Studies Depression Scale
COPD: Chronic obstructive pulmonary disease
DBS: Dried blood spot
HbA1c: Hemoglobin A1c
HDL-C: High-density lipoprotein cholesterol
HRs: Hazard ratios
HRS: Health and Retirement Study
HsCRP: High-sensitivity C-reactive protein
SDs: Standard deviations
TC: Total cholesterol
