# Supplementary material for: Associations of plasma high-sensitivity C-reactive protein concentrations with all-cause and cause-specific mortality among middle-aged and elderly individuals
Source: Immun Ageing. 2019 Nov 5;16:28. doi: 10.1186/s12979-019-0168-5 (PMC6833146; doi:10.1186/s12979-019-0168-5)
Supplement: Supplementary file 1 — Additional file 1: Figure. S1. Flowchart of the participant enrolment. Table S1. Role of potential mediators in explaining the association between each 1 mg/L increase in the hsCRP concentration and all-cause, cardiovascular and cancer mortality. Table S2. Multivariable hazard ratios (HR [95% CI]) of mortality by quartiles of high-sensitivity C-reactive protein after excluding deaths during the first two years of follow-up. Table S3. Multivariable hazard ratios (HR [95% CI]) of mortality by tertiles of high-sensitivity C-reactive protein. Table S4. Multivariable hazard ratios (HR [95% CI]) of mortality by quintiles of high-sensitivity C-reactive protein. Table S5. Multivariable hazard ratios (HR [95% CI]) of mortality by clinical categories of high-sensitivity C-reactive protein. [file 12979_2019_168_MOESM1_ESM.docx]

**
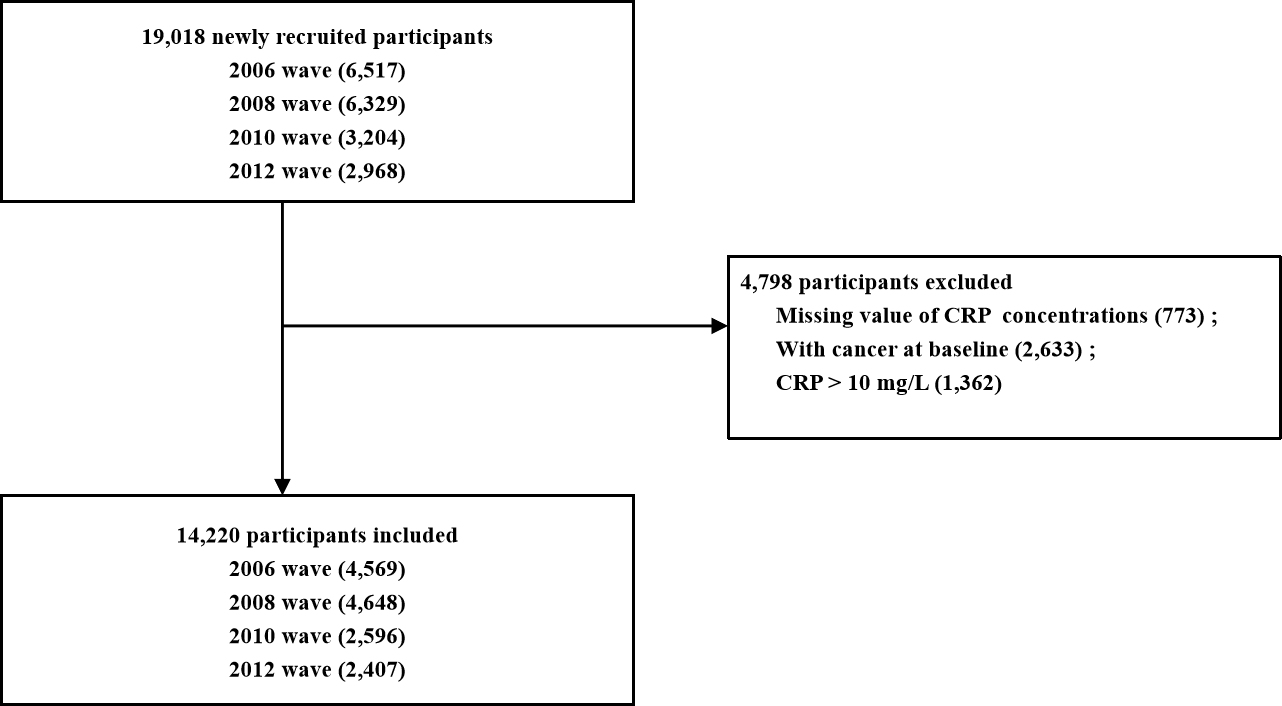
**

Figure S1. Flowchart of the participant enrolment

**Additional file**

**Table S1.** Role of potential mediators in explaining the association between each 1 mg/L increase in the hsCRP concentration and all-cause, cardiovascular and cancer mortality

| Model | HRs (95% CI) of mortality | | | | |
| --- | --- | --- | --- | --- | --- |
|  | All-cause mortality |  | Cardiovascular mortality |  | Cancer mortality |
| Model 2**^†^** | 1.08 (1.05-1.10) |  | 1.06 (1.02-1.10) |  | 1.10 (1.05-1.15) |
| Model 2+ hypertension | 1.07 (1.05-1.10) |  | 1.06 (1.02-1.09) |  | 1.10 (1.05-1.15) |
| Model 2+ diabetes | 1.08 (1.06-1.10) |  | 1.06 (1.03-1.10) |  | 1.10 (1.06-1.15) |
| Model 2+ heart disease | 1.08 (1.06-1.10) |  | 1.06 (1.03-1.10) |  | 1.10 (1.05-1.15) |
| Model 2+ stroke | 1.08 (1.05-1.10) |  | 1.06 (1.02-1.10) |  | 1.10 (1.05-1.15) |
| Model 2+ psychological problems | 1.08 (1.05-1.10) |  | 1.06 (1.02-1.10) |  | 1.10 (1.05-1.15) |
| Model 2+ pulmonary disorders | 1.07 (1.05-1.09) |  | 1.06 (1.02-1.09) |  | 1.10 (1.05-1.15) |
| Model 2+ ADL | 1.08 (1.06-1.10) |  | 1.06 (1.03-1.10) |  | 1.10 (1.05-1.15) |
| Model 2+ CES-D 8 | 1.08 (1.05-1.10) |  | 1.06 (1.02-1.10) |  | 1.10 (1.05-1.15) |
| Fully adjusted model**^‡^** | 1.07 (1.05-1.10) |  | 1.06 (1.02-1.10) |  | 1.10 (1.05-1.15) |

8-question Center for Epidemiologic Studies Depression Scale: (CES-D 8);

**^†^** adjusted for age, sex, race, educational level, current smoking status, alcohol consumption, regular exercise, body mass index (BMI), household income, total cholesterol (TC) concentration, high density lipoprotein-cholesterol (HDL-C) concentration, and hemoglobin A1c (HbA1c);

**^‡^**model 2 plus adjusted for CES-D 8 score, hypertension, heart disease, stroke, cancer, diabetes, pulmonary disorder, psychiatric problems, and limitations in activities of daily living (ADLs).

**Table S2.** Multivariable hazard ratios (HR [95% CI]) of mortality by quartiles of high-sensitivity C-reactive protein after excluding deaths during the first two years of follow-up

| HsCRP quartiles (mg/L) | All-cause mortality |  | Cardiovascular mortality |  | Cancer mortality |
| --- | --- | --- | --- | --- | --- |
| Q1 (<0.76) | 1.00 (reference) |  | 1.00 (reference) |  | 1.00 (reference) |
| Q2 (0.76-1.50) | 1.03 (0.88-1.21) |  | 1.10 (0.84-1.44) |  | 1.02 (0.72-1.44) |
| Q3 (1.51-2.76) | 1.08 (0.92-1.27) |  | 1.11 (0.84-1.47) |  | 1.15 (0.81-1.63) |
| Q4 (>2.76) | 1.42 (1.21-1.65) |  | 1.37 (1.05-1.80) |  | 1.63 (1.16-2.28) |
| *P* for trend | <0.001 |  | <0.001 |  | <0.001 |

**^†^** Adjusted for age, sex, race, educational level, current smoking status, alcohol consumption, regular exercise, body mass index (BMI), household income, total cholesterol (TC) concentration, high density lipoprotein-cholesterol (HDL-C) concentration, and hemoglobin A1c (HbA1c).

**Table S3.** Multivariable hazard ratios (HR [95% CI]) of mortality by tertiles of high-sensitivity C-reactive protein

| HsCRP tertiles (mg/L) | All-cause mortality |  | Cardiovascular mortality |  | Cancer mortality |
| --- | --- | --- | --- | --- | --- |
| Q1 (<1.14 mg/L) | 1.00 (reference) |  | 1.00 (reference) |  | 1.00 (reference) |
| Q2 (1.14-2.78 mg/L) | 1.13 (1.00-1.28) |  | 1.21 (0.99-1.49) |  | 1.18 (0.90-1.55) |
| Q3 (>2.78 mg/L) | 1.42 (1.26-1.60) |  | 1.46 (1.19-1.79) |  | 1.49 (1.14-1.95) |
| *P* for trend | <0.001 |  | <0.001 |  | <0.001 |

**^†^** Adjusted for age, sex, race, educational level, current smoking status, alcohol consumption, regular exercise, body mass index (BMI), household income, total cholesterol (TC) concentration, high density lipoprotein-cholesterol (HDL-C) concentration, and hemoglobin A1c (HbA1c).

**Table S4** Multivariable hazard ratios (HR [95% CI]) of mortality by quintiles of high-sensitivity C-reactive protein

| HsCRP quintiles (mg/L) | All-cause mortality |  | Cardiovascular mortality |  | Cancer mortality |
| --- | --- | --- | --- | --- | --- |
| Q1 (<0.71) | 1.00 (reference) |  | 1.00 (reference) |  | 1.00 (reference) |
| Q2 (0.71-1.37) | 1.063 (0.90-1.24) |  | 1.12 (0.85-1.47) |  | 1.04 (0.72-1.50) |
| Q3 (1.38-2.31) | 1.11 (0.95-1.30) |  | 1.18 (0.90-1.55) |  | 1.25 (0.88-1.77) |
| Q4 (2.32-4.17) | 1.29 (1.11-1.51) |  | 1.40 (1.07-1.82) |  | 1.36 (0.96-1.93) |
| Q5 (>4.17) | 1.54 (1.32-1.79) |  | 1.57 (1.21-2.04) |  | 1.72 (1.22-2.43) |
| *P* for trend | <0.001 |  | <0.001 |  | <0.001 |

**^†^** Adjusted for age, sex, race, educational level, current smoking status, alcohol consumption, regular exercise, body mass index (BMI), household income, total cholesterol (TC) concentration, high density lipoprotein-cholesterol (HDL-C) concentration, and hemoglobin A1c (HbA1c).

**Table S5** Multivariable hazard ratios (HR [95% CI]) of mortality by clinical categories of high-sensitivity C-reactive protein

| HsCRP categories (mg/L) | All-cause mortality |  | Cardiovascular mortality |  | Cancer mortality |
| --- | --- | --- | --- | --- | --- |
| <1 | 1.00 (reference) |  | 1.00 (reference) |  | 1.00 (reference) |
| 1-3 | 1.12 (0.99-1.27) |  | 1.18 (0.96-1.46) |  | 1.24 (0.94-1.63) |
| >3 | 1.45 (1.28-1.65) |  | 1.44 (1.16-1.78) |  | 1.51 (1.13-2.01) |
| *P* for trend | < 0.001 |  | < 0.001 |  | < 0.001 |

**^†^**Adjusted for age, sex, race, educational level, current smoking status, current drinking status, regular exercise, body mass index (BMI), household income, total cholesterol (TC) concentration, and high density lipoprotein-cholesterol (HDL-C) concentration.
